# Outlines of Human Pathology

**Published:** 1837-01

**Authors:** 


### ART. III.

*Outlines of Human Pathology.* By HERBERT MAYO, F.R.S. &c., Professor of Anatomy, Physiology, and Pathological Anatomy, in King's College, London; Surgeon to the Middlesex Hospital.—*London*, 1836. 8vo. pp. 595.

THE plan of this work differs considerably from that of others in our language, bearing the same or a similar title. The "Elements" of Dr. Parry, the "Principles" of Dr. Pring, and the "Outlines" of Dr. Alison are chiefly occupied with enquiries into the general laws of morbid phenomena, without any further reference to the lesions of particular organs than was thought necessary to the purposes of illustration; but Mr. Mayo's treatise is a summary recital of the changes in the several parts of the body, and of their symptoms. The absence of etiology and therapeutics, at least as constituent parts of the whole work, distinguishes it from our systems of practical medicine and surgery; and in this respect, if we do not mistake, it stands almost alone, or at all events has had no predecessors of importance. The French have many works devoted to pathology and diagnosis only; but it would seem to have been always difficult to our countrymen to divest their minds of the practical applications of knowledge; and we confess that we should be sorry if they lost this their predominant attachment to objects of immediate utility.

We cannot say that we approve of the arrangement which the author has thought proper to adopt in the treatment of his subjects. It may be urged indeed that this is a matter of but slight moment, and that it signifies little whether the nature of inflammation and carcinoma is discussed before or after the account of periostitis and osteo-sarcoma; an opinion which we should not perhaps dispute, if the reader were supposed to possess a general acquaintance with the topics, and to look to the work merely for new facts and speculations. If he is already familiar with the elements of morbid changes, it matters not how composite may be the diseased structure first submitted to his attention, and it is even doubtful whether the former need be introduced into the work at all. But Mr. Mayo's "Outlines" are designed quite as much for students as for proficients, and therefore we contend that the order of the topics ought to have been from the simple to the more complex. If the author, again, had limited himself to the description of the alterations in the organs, without noticing the mode of their production, it would have been quite indifferent where he commenced: but his design was not so scanty; even from the commencement he attempts, and often with good success, to unravel the web of disease, though the texture of the threads must remain a mystery till the reader has advanced pretty far into the volume.

In all ages it has been the object of medical speculators to resolve the multifarious phenomena of disease into a few elementary forms, an object no less desirable in a practical than in a philosophical point of view. Some of the alleged pathological elements have been mere hypothetical ingredients of morbid changes, and others have been nothing more than certain characters common to the manifestations of disease in different parts. Strictum and laxum, sthenia and asthenia, illustrate the latter; while the former may be exemplified by the fancied depravations of humours, the supposed collisions between molecules of different shapes, and the unseen spasm of capillaries. The medical philosophy of later years acknowledges neither symptoms nor imaginary states of the solids and fluids, as the components of disease; it allows of none but such as are demonstrable by anatomical or chemical analysis, though it confesses that there are but too many specimens of disease, the analysis of which is at present impracticable. Strictly speaking, not a single morbid *element* has as yet been discovered, though we have certain *proximate principles*, such as inflammation, hypertrophy, tubercle, cancer, &c. These may have their seat in organs very distant from each other, and differing greatly in texture, but they have characters which enable us to recognize them wherever found, and however modified by their locality. We are of opinion that the student should acquire a knowledge of these simpler forms, before he is introduced to the combinations in which they occur in the lesions of particular organs. How little prepared is he for understanding the complicated alterations in chronic peritonitis, for example, unless previously instructed in the nature of inflammation and tuberculous deposition! How can he appreciate the difference between the cadaveric redness of mucous membrane, and the marks of true gastritis, if he has previously heard nothing of congestion as distinguished from inflammation?

These very obvious considerations, we think, might have alone been sufficient to deter Mr. Mayo from the plan followed in his work. It is indeed surprising that, accustomed as he is to view subjects in their philosophical relations, he should have adopted an arrangement based as little upon anatomical and physiological, as upon pathological principles. Treatises upon special anatomy very properly commence with the bones and muscles, because the situation of parts respectively to each other is the principal object of instruction, and, as those organs occupy the greatest space in the fabric, they are the most convenient objects of reference. It would be no less absurd to define the course of rivers and roads, and the situation of towns in a country, before describing the country itself, than to attempt to point out the direction of arteries and nerves, and the locality of viscera, before the parts traversed by the former and containing the latter have been made known. But the position of organs is of comparatively small concern to pathological anatomy, which regards the parts individually, and with reference to their structure, and therefore might be expected to commence its researches in the tissue which is most widely diffused, and indeed essential to the very existence of every part of the frame. Whatever other tissues enter into the composition of a part, they must derive their support from, and throw their incumbrances upon the capillary system, and their condition is rarely altered without implicating this system, if not actually owing their changes to it. Every disease which leaves traces in a part leaves them in the quantity or quality of the blood, in the nutritive, secretory, and absorbent processes; and, with these textural conditions and processes, what part is more intimately connected than the capillary system? Here then, we think, there is every reason for commencing pathological enquiries; and here, accordingly, we shall commence our survey of Mr. Mayo's labours. Only seven pages and a half are devoted to diseases of the capillaries; certainly not a very liberal allowance to such subjects as Anæmia, Hyperæmia, Inflammation, and the production of Serum, Lymph, and Pus. Although the reader may be contented with learning just as much as has been absolutely ascertained upon these matters by observation, without engaging in speculative controversies, and may not expect that their phenomena should be viewed in relation to other parts of the system, he will yet be disposed to wish that this section had been rather more ample.

The following is all that is said of hyperæmia.

" *Hyperæmia*, or congestion. The minute vessels may be unusually distended with blood, either through obstruction upon the veins—as in a limb to which a bandage is applied previously to venesection—or through strong action of the heart, as upon the skin during the hot stage of inflammatory fever; or, finally, through a change in the state of the capillaries, enabling the blood to enter them with greater facility and in greater quantity than before. The cases in pathology in which the vessels display a lowered tone, or yield a freer passage than natural to the blood, are referrible to three heads. 1. Irritation, as, for instance, in the suffusion of the conjunctiva, when a foreign body gets within the eyelids, or in the state of the mucous membrane of the small intestines in Asiatic cholera. 2. Vascular nævi, or, as they are often termed, erectile tissue. 3. Some kinds of malignant tumours." (P. 426.)

In an elementary work upon pathology, we have good reason for complaint if the author does not explain the meaning which he puts upon such a word as irritation, especially when so large a class of disorders has been referred to it by some writers. Andral is for banishing inflammation from our vocabulary; we should certainly be heartily glad to get rid of irritation, the undiscriminating use of which in medical treatises has often produced in our minds the *feeling* which the word denotes, when it has failed to summon up any distinct *idea*. How can the student escape perplexity when sometimes it would appear to signify that congested state of the capillaries which is immediately traceable to an external agent, as a poison, or a chemical or mechanical injury; sometimes a mere derangement of sensation; sometimes a slight shade of inflammation, as in the complications of febrile diseases; sometimes a state antecedent to but not constituting congestion, as in the old saying "ubi irritatio, ibi affluxus;" sometimes a condition of the nervous system incident to hysterical, neurotic, and debilitated subjects; sometimes an exalted vitality, as in the writings of the physiological pathologists; and sometimes is the synonym of simple vital action, correlative with the irritability of Glisson? Let us suppose a student reading the passage above cited. Those species of congestion which are occasioned by obstruction and by an excessive supply of blood from the central organs of circulation, will be intelligible enough; and when he comes to the description of that state in which the vessels "display a lower tone or yield a freer passage than natural," a state which will be at once perceived to be a matter of inference only, because he has learnt in a preceding page that the very existence of such vessels is only inferred, (like the ultimate fibre of Haller, "invisibilis est ea fibra, mentis solâ acie distinguitur,") he will be prepared for the absence of any account of the mechanism whereby this change in the vessels is effected, and for finding nothing more than a classification of the specimens of it. Of the three heads of arrangement, irritation he finds is the first. Meeting with no explanation of this term, and perhaps even ashamed of having no definite idea connected with a word in such common use, he instinctively passes on to the exemplification, and is gratified to find it of so palpable a nature as the redness of the conjunctiva provoked by a foreign body. But his satisfaction is short-lived when he arrives at the next instance, viz. "the state of the mucous membrane of the small intestines in asiatic cholera." *Ignotum per ignotius,*—he turns back to the section on the pathology of the alimentary canal, where he finds it laid down that an invariable appearance in the bodies of persons who have died of cholera, is a pink coloration of the mucous membrane of the intestines, equally distinguishable from the redness of inflammation and the "dark vascularity" characteristic of venous congestion. But still he is at a loss to perceive what resemblance this hyperæmia bears to that produced by a foreign body applied to the conjunctiva. We fear that his difficulty will scarcely be cleared up by reading the following sentence. "When the intestines have been under the *irritation* of that disorder (diarrhœa) for some hours, they have directly passed into the state of cholera." Is it meant that the diarrhœa irritates the intestines? Scarcely; since the diarrhœa is itself a disorder of the intestines. Does it mean that the irritation which causes or coexists with the diarrhœa, passes into the state of cholera? If so, the student having learnt that the state of the intestines in cholera is an instance of irritation, learns in addition by his reference that it is a species of congestion, and that the irritation of diarrhœa passes into the irritation of cholera; but he still remains in the dark as to the nature of irritation, and consequently as to the analogy between the hyperæmia of an irritated conjunctiva, and the hyperæmia of the intestines in cholera. True, they are said to be instances of hyperæmia caused by a change in the capillaries themselves, but so are "vascular nævi," and "some kinds of malignant tumours."

The omissions in the above passage are scarcely less striking than the obscurity arising from the use of an undefined term. To which of the three heads are we to assign the congestions occasioned by gravitation, and by the sedative influence of cold, or those which occur in scorbutus, typhus, and other disorders accompanied by increased fluidity of the blood?

" *Inflammation.* In inflammation there is an afflux of blood to a part, and something more. Not only are the minutest vessels in the inflamed part larger than before, and hold more blood, but the blood which they contain, in some is perfectly stagnant, in others moves very slowly; and where it is stagnant, or nearly motionless, is changed in quality, the distinction of a transparent liquid and particles suspended in it being lost. These appearances, which have been observed by many, I have repeatedly produced by irritating the membrane of the foot of frogs under the microscope." (P. 426.)

The author has here exhibited very clearly and forcibly the true nature of inflammation. We do not blame him for having avoided the controversy respecting increased or diminished action, which may perhaps be considered now at rest. The advocates of increased action grounded their theory upon what was accidental rather than essential to the inflammatory state; viz. the throbbing arteries, the increased heat, and the swelling of surrounding parts, phenomena which certainly prove an increased action, but not in the vessels under dispute. The broad facts of inflammation are certainly the stagnation of the blood, and the consequent alteration of its qualities, but we could have wished that Mr. Mayo had pointed out some of the instances in which it is difficult to determine whether they ought to be referred to mere congestion or to inflammation. The same lesion is not unfrequently designated by one observer as congestion, and by another as inflammation, because the former limits his idea of inflammation to a condition in which there is not only an inordinate quantity of blood, but in which certain products have been separated; while the latter regards an accumulation of blood, provided it does not clearly result from venous obstruction, a sufficient proof of inflammation. The student should have been instructed in the discrimination of the epithets *active* and *passive* congestion, the former being confined to congestion produced by arterial plethora, the latter to that resulting from venous plethora, or from a preternatural fluidity of the blood, or as some suppose from debility in the extreme vessels. Active and passive inflammation, agreeing in the changes of the blood, but differing as to the condition of the neighbouring parts, and the disturbance of the general system, should have been at least noticed; also the chronic species, as differing not only in the time occupied, but likewise in the nature of the products.

We find no mention, either in the present section or throughout the work, of what is called specific inflammation. No notice is taken of the different phenomena of inflammation according to the part invaded, or how the composition and texture of the latter may influence the tendency of the process to spread, to subside, and to afford particular products. In no part of the book is there any notice of mucous or of serous inflammation, as such, though inflammations of particular portions of mucous and serous membranes are of course described under the pathology of the organs in which they occur.

Besides these omissions of an anatomical character, we might complain of the absence of any remarks upon the connexion of the symptoms of inflammation on the one hand, and of congestion on the other, with the physical alterations, or upon the difference between the symptoms of each of these states, or upon the different conditions of the whole system in which they respectively occur. Space has been found for the semeiology of a great number of diseases, and many pages are occupied with histories of cases, some of which might very well have given place to observations which would have been more in keeping with such a work as "Outlines of Pathology," and which the author was very capable of writing.

The circumstances under which serum is separated from blood while circulating, are very ably described. In barely more than a page we find condensed nearly all that is certainly known of the pathology of dropsy. We cannot forego the pleasure of presenting our readers with this passage.

"1. *a.* The most frequent cause of effusion of serum is retardation of the progress of the blood in the veins. The effect in this instance is perhaps entirely of a mechanical nature. The capillaries being gorged, the thinnest part of the blood transudes through their coats. When the axillary vein is compressed by enlarged and indurated lymphatic glands, serum thus escapes into the cellular membrane, or the limb becomes œdematous. When the upper vena cava is obliterated by inflammation, or obstructed by the pressure of a tumour, the face, throat, and arms are loaded with anasarca. When the inferior cava is obstructed, the legs become anasarcous. When the cavity of the femoral vein is obliterated by inflammation, the same result takes place. When the hepatic circulation is obstructed by thickening of Glisson's capsule, abdominal dropsy, or ascites, follows. When the inferior cava is compressed near the diaphragm, by induration of the pancreas or adjacent lymphatic glands, dropsy with anasarca of the legs ensues. When there is valvular disease of the heart, allowing the stroke of the left ventricle to tell backwards and impede the propulsion of blood through the lungs, and even its passage through the right side of the heart, the lungs and the whole body are liable to become infiltrated with serum. Finally, the pressure of ovarian tumours, or of dropsy, on the iliac veins, will render the legs œdematous.

"*b.* It is probable there may be a condition of the blood—such as, for instance, extreme attenuation from repeated hemorrhage—which may dispose it to part with serum readily, and so to give rise to œdema. A thin blood and a weak heart are capable of occasioning anasarca and dropsy.

"*c.* Chronic inflammation of the kidney, impeding the secretion of urine, may be productive of anasarca. This is one of the causes of the anasarca which follows scarlatina. Dr. Bright discovered and described the influence of this cause in determining effusion in persons who have lived intemperately; and showed its connexion with albumen in the urine.

"*d.* General effusion of serum partakes sometimes of an inflammatory character. In young and not otherwise unhealthy persons, œdema of the legs, of the arms, and face, with ascites, will sometimes supervene after exposure to cold, being attended with slight tenderness of the swollen parts, and often with albuminous urine.

"*e.* Serous effusion is a constant attendant of ordinary inflammation, exuding equally into the interstitial cellular membrane, into the cellular tissue of organs, upon serous and mucous surfaces, and upon the skin. Its source in these instances is again probably mechanical, and attributable to the obstruction of blood in the inflamed capillaries. The softening common to all fleshy textures at the outset of acute inflammation is in part at least produced by their infiltration with serum." (P. 427.)

The following is the sum total of what the author thinks necessary to say upon Hunter's adhesive inflammation.

"Lymph, in its chemical composition, resembles coagulated albumen or the fibrin of the blood. It is probably the latter element exuding in a liquid state, and then coagulating. The colourless part or size, which in two or three minutes separates and floats upon the surface of liquid inflamed blood, Hewson has shown to be *fibrin unusually attenuated;* but if there exists in inflammatory blood a portion of unusually attenuated fibrin, and if that fibrin can be shown to have a disposition to separate from the rest of the blood, and if its tenuity would render its mechanical escape from the containing vessels the more facile, and if from vessels in inflamed parts a liquid fibrin is found to escape, we seem to be already in possession of all the elements of this phenomenon." (P. 428.)

We are scarcely satisfied with this somewhat off-hand mode of treating such a subject. Is this attenuated state of the fibrin placed beyond doubt? Do not many physiologists maintain that there is a stronger aggregation between the particles of inflammatory than of ordinary fibrin? But, without disputing the truth of the facts thus assembled, we cannot agree with the author in considering them to be *all* the elements of the process in question, so long as we are ignorant whether inflammatory blood taken while flowing from a vein is identical in quality with blood stagnant in inflamed capillaries, or so long as it remains unexplained why lymph is sometimes effused in preference to serum, though the latter has greater tenuity, and consequently greater facility of escape, and why this process occurs more readily in some tissues than others. We should have been glad to have known whether Mr. Mayo believes that the difference in the liability of tissues to this occurrence is as great as has been generally represented. To us it has appeared doubtful whether the difference is not rather apparent than real; whether in fact the tendency to effusion of lymph is not more equal than has been supposed, while the *traces* of the effusion are more conspicuous and permanent in some parts than in others, in consequence of their mechanical relations, and the nature of their normal secretions. Is it improbable that fibrine is separated as frequently on mucous as on serous membranes, but that it escapes observation partly because it is confounded with mucus, partly because it is removed with other substances that traverse these membranes, and partly because *adhesion*, one of the strongest evidences of the effusion, occurs with difficulty in parts where the contact of opposite surfaces is infrequent, and at all events subject to continual interruption? The portions of the mucous system in which we trace the effects of fibrinous secretion are precisely those in which either the agents that remove the secretion are feeble, or the surfaces approach each other very closely. Such are the adhesions and obliterations of the lachrymal and biliary ducts, the ureters, and the urethra, and the consolidation of the pulmonary tissue by coagulable lymph effused in the air-cells. Lymph may not only be effused upon mucous surfaces, but may become organized as upon serous; examples of which, had they been duly examined, would not have been so frequently mistaken as they have been, for detachments of mucous membrane.

The formation of pus is treated of rather more at length than the last subject. After noticing the similarity in form between the globules of pus and those of blood, and the greater size of the latter, the author introduces an extract from M. Gendrin's treatise, containing the celebrated observation of the process of suppuration in a frog's foot placed under the microscope, and concludes the section with the following passage.

"The observations which I have quoted from M. Gendrin, when taken in connexion with the facts previously stated, simplify the theory of inflammation, and satisfactorily explain the alliance of all its leading phenomena. The initiatory effusion of serum and of lymph, dependent upon the visible obstruction of the circulation—the lymph the same substance with attenuated inflammatory fibrin—the consequent occasional mixture of blood with lymph—the formation of pus secondary to and later than the secretion of serum and lymph, being the result of protracted inflammatory action—the solid particles in pus, although larger, yet of the same remarkable figure with those of the blood, and doubtless the same enlarged—the occurrence of blood in pus mixed with it in streaks, or generally diffused through it—the organization of lymph by extension of vessels, some at first containing pus, others a thick red liquid; and lastly, inflammatory gangrene proceeding from the vessels being in certain cases irrecoverably obstructed, are phenomena, which may be declared to be now grouped under one law." (P. 431.)

It cannot be denied that this grouping of the phenomena is executed in a very masterly manner; but we regret that in his anxiety to present a connected view of the facts the author should have omitted one which is certainly of some importance, we mean *ulceration*. Was it that he found some difficulty in exhibiting the relation of this phenomenon to the rest? A person moderately acquainted with the subject, would discover hints of the possibility of such a change in certain parts of M. Gendrin's description, in such phrases for instance as "the eschar begins to detach itself," "solution of continuity;" but whether a student would identify these changes with ulceration is more doubtful perhaps, than whether he would recognize in "the organization of lymph by extension of vessels" the familiar process of granulation. Ulceration having been particularly associated with absorption since the time of J. Hunter, we thought it just possible that the author might have chosen to treat of it under the absorbent system, or the veins, but in these and other parts of the work our search was fruitless. If the reader wishes to know something more of gangrene than is contained in one of the clauses of our last quotation he will be disappointed; but why it was less incumbent on the author to enumerate the different circumstances under which gangrene takes place, than those which influence the effusion of serum, we do not profess to understand.

We have thought it requisite to notice these omissions; but, that we may not be deficient in justice, we shall here introduce a short extract from the introduction to the work.

"In the present treatise, nothing so ambitious has been attempted as a complete discussion of the subjects above enumerated. The author's aim has been, to arrange in a perspicuous order, and to describe with as much brevity as is consistent with clearness, the morbid affections to which the different organs of the human body are subject. This, with some accidental omissions, and with the intentional omission of those subjects which are commonly made isolated studies, he trusts has been accomplished to an extent that may render the following pages not without their use." (*Introduction*, p. xxvii.)

Whether the omissions which we have pointed out belong to the accidental or the intentional, it may be difficult for the reader to determine.

In accordance with what we have stated to be our opinion respecting the proper order of subjects, we shall now, having considered Mr. Mayo's remarks upon inflammatory products, pass on to the other morbid deposits. Tubercle is discussed under the diseases of the lungs. The account given of this substance is brief, but comprehends nearly all its principal characters, and the more important of the changes which it undergoes. The author evidently follows M. Andral very closely, but, when he says that the deposition "is unattended by symptoms of general or local inflammation," he removes farther from the doctrines of the physiological school, than even that eclectic pathologist who concludes his admirable chapter on tubercle with the following words: "dans l'état actuel de la science le tubercule doit être considéré comme le resultat d'une modification ou perversion de sécrétion, que précède ou accompagne souvent une congestion sanguine active."* If we state that Mr. Mayo believes tubercle to be unorganized, that it is softened by mere infiltration with fluid from the tissue where it exists, and that the grey granulations found in the lungs are not tuberculous, we have given his opinions on the points which have been most disputed; but, with regard to the last mentioned of these, we doubt whether the most experienced pathologists of the day would agree with him in saying that Andral has *proved* that the grey bodies in question do not lead to true tubercles, and that they are the effects of inflammation in a few of the air-cells. That these formations are limited to tuberculous subjects, and that they become opaque and identical with tuberculous matter, was taught by Laennec, and is maintained by Louis. In one of his most recent publications the latter author, when speaking of the uniform complication of tubercles with chronic ulceration of the small intestines, declares, "je n'ai pas vu un seul sujet emporté par une maladie chronique, et ayant des ulcerations dans l'intestin grêle, qui n'eût *des tubercules dans les poumons, ou des granulations grises demi-transparentes, ce qui est la même chose.*"† Dr. Carswell has explained the discrepancy among anatomists upon this subject, by shewing that tuberculous matter is often mixed with the natural and morbid secretions of the part; thus in the lungs the semitransparent appearances may be due to the admixture of inspissated mucus; and, in the peritoneum, to the presence of coagulable lymph.

Directed by a passage in the Introduction, we turned to Diseases of the Breast for the author's views of Carcinoma, but were disappointed to find that he had thought proper to avoid any general discussion of this formation; his reason for the omission being that Mr. Kiernan's researches have not yet been published. We entertain the greatest respect for the powers and performances of that

* Precis d'Anat. Pathol. I. 438.

† Examen de l'Examen de M. Broussais, p. 18.

excellent anatomist, and promise ourselves a great increase of pathological knowledge by the continuance of his labours; but in the mean time it is not unreasonable to ask whether Laennec, Abernethy, Burns, Cruveilhier, Andral, and Carswell, have done nothing towards defining the characteristics of carcinomatous disease, or in pointing out the similarity of its progress in different parts, its modifications in form and consistence by tissues and organs, its arrangement, the localities which it prefers, &c.? Unless Mr. Mayo thinks (and he tells us that he is "in some degree acquainted with Mr. Kiernan's unpublished views,") that students would have by and bye to *unlearn* what has been taught by the pathologists enumerated, he ought surely to have made them acquainted with the progress already made in this investigation. Instead however of any such treatment of the topics as we had reason to expect, we were obliged to content ourselves with the external characters, and the general anatomical appearances of carcinoma as observed in the breast.

"This disease, which forms forty-nine fiftieths of malignant diseases of the breast, when examined after death, presents three distinct morbid appearances, which are often met with combined in the same part, although sometimes, but rarely, they are met with separately. *a.* A tumour, the whole of which nearly resembles cartilage in structure, dense, of a greyish or blueish white, with a slight approach to transparency, elastic, cutting with the same sound as cartilage. *b.* A tumour, cutting crisply, like the texture of an unripe pear, of a grey colour, succulent, with yellowish or whitish lines, containing an inspissated substance, probably tubuli lactiferi. *c.* A softer texture, more vascular, or red with partial vessels, and succulent, not without some crispness when cut through, when squeezed giving out fluid partly serous, partly opaque and white." (P. 566.)

Some cases are related illustrative of this distinction, to which succeeds an excellent account of the progress of the disease in the mammæ. The remarks upon an important practical question are very judicious.

"After amputations of a scirrhous breast under the most favorable circumstances, that is to say, when the operation is performed at the earliest period at which the structural character of the disease has declared itself in the gland, no other part being yet invaded by it, and the diseased structure being entirely removed; I believe, that, in ninety-nine cases out of a hundred, the disease returns either in the cicatrix, or in the axillary or subclavian glands. The operation therefore cannot be performed with any reasonable prospect of saving the patient eventually from the disease. But the period of the return of scirrhus varies from six months to two or three years, or even longer. The interval may be one of health and hope; and even when the disease reappears, it does not in general return in a character of such formidable suffering as in its ordinary course it presents. The worst suffering in cancer arises from the destructive ulceration of the original scirrhus: the changes which supervene in the secondary scirrhus, whether in the cicatrix or the lymphatic glands, are generally mild, compared with the former. The constitution likewise sinks more rapidly on the return of the disease, being by that time more completely undermined than at the first invasion of scirrhus." (P. 573.)

We now turn to the pathology of the blood. Upon this subject we are not presented with any original information; we do not however mention this by way of complaint, but rather because the introduction of observations peculiar to himself would have been an excuse for the defective manner in which the author has compiled this section. We have been struck on more than one occasion by the implicit reliance which he places upon some one authority, and have already noticed his passing by a whole host of distinguished pathologists, and leaving a subject all but untouched, because a favorite investigator has not laid it before the public. In another place we find him delivering doctrines on the faith of a single individual, without hinting that many able investigators have formed opinions of a very different nature.

"*Inflammatory blood* is characterized by a colourless *crust* or *size* upon the upper surface of the clot, which in the acutest inflammation is of the depth of a quarter to a third of an inch, and is strongly contracted or cupped.

"The principal facts which are known upon this subject, were observed by Mr. Hewson. They are the following:—1. In inflammatory blood the process of coagulation is slow, but the red particles begin to subside immediately, as may be seen in the blueish cast which the surface assumes, from which a transparent liquid can be taken that coagulates. 2. The subsidence of the red particles depends upon attenuation of the fibrin. In two phials, one of inflammatory serum, one of uninflammatory serum, a teaspoonful of serum loaded with red particles (from uninflammatory blood) was poured. They subsided with equal slowness in both. Two teaspoonfuls, one of serum with red particles from inflammatory blood, one of serum with red particles from uninflammatory blood, were poured into two phials of uninflammatory serum. There was no difference in the time of the subsidence of each. 3. The disposition to form a size is capable of being modified in a very brief period, by the condition of the nervous system." (P. 481.)

We cannot be accused of hypercriticism in pronouncing this to be a most unsatisfactory statement. Were it true, sad indeed would have been the torpor of modern science to have done nothing for the elucidation of the subject since the time of Hewson. But, while glad to believe it to be untrue, we regret that the knowledge of the profession upon this subject should be so stated. We suspect that some readers might find a little difficulty in ascertaining the drift of the experiments related; but they must conclude that the chief meaning of these "principal facts" is, 1st, that inflammatory blood coagulates slowly, but the slowness is not the cause of the subsidence of the red particles, because they may be seen to separate immediately; 2dly, that their subsidence is occasioned by the attenuation of the fibrin, because they subside just as soon in inflammatory as in uninflammatory serum. This is, we suppose, "an inference by exclusion;" the time of coagulation has nothing to do with the fact in question, nor has the serum; ergo, an alteration in the specific gravity of the fibrin must be the cause. Nothing is said of the opinion that the separation of the red particles from the fibrin is independent of gravitation; and yet we know that very thin films of blood will shew these constituents separate, but arranged laterally instead of in an upper and lower lamina. This may be often noticed on the blade of a lancet, which, if the blood is inflammatory, will present a speckled appearance, caused by the red particles and the transparent fibrin. Dr. Alison has been led by this and other facts to assign the phenomenon to a vital repulsion between the particles. Whether this is the true doctrine, or Dr. Babington's notion that the longer fluidity of the *liquor sanguinis* gives time to the insoluble red particles to subside, we have not time to enquire; but certainly we dissent from the opinion that the principal facts respecting the inflammatory crust are contained in the above quotation.

The author cites from Hewson the case of a young man who was bled "in an attack of inflammatory fever:" out of four cups abstracted, size was formed in the third only. At the beginning of the operation the patient was faint by fear, at the end by loss of blood. This case illustrates very well the influence of the nervous system, which some would explain by saying that the coagulation was accelerated under this agency; others by supposing that in the first cup there was a loss of vital repulsion between the particles, but that in the fourth the increased quantity of serum had favoured coagulation. Mr. Mayo would, we apprehend, attribute the want of the crust to the density of the fibrin; but no attempt is made to explain how this view may be reconciled with the statement that the size in the third cup, though it continued for a long time fluid, was afterwards found "very thick and tough."

Nothing is said of the strong aggregation of the fibrin, or of its proportionate quantity, or of that of the albumen. Nor is the effect of the shape of the receiver or of the orifice adverted to; facts not less familiar than the influence of faintness, but not very easy to explain. Exposure to the air, which is known to promote coagulation, may account in some measure for the difference. Blood received into a large flat vessel or drawn in a small stream has a greater quantity exposed to the air in a given time; but it must be remembered that coagulation is favoured by extension over a wide surface even in vacuo.

The observations upon the state of the blood in disease of the kidney with albuminous urine, in diabetes, cholera, fever, and jaundice, are valuable, and are acknowledged to be derived from Dr. Babington's excellent article in the Cyclopædia of Anatomy. But why should the condition of the blood in rheumatism, gout, and scorbutus, have been omitted, even if the author did not think it worth while to speak of its changes in those states of the system which lead to malignant diseases?

In proceeding to examine with the author some of the maladies of particular organs, we must leave almost unnoticed the very interesting section that treats of diseases of the heart, as we have elsewhere dwelt largely on this subject. We cannot, however, forbear remarking that we observe here also many important omissions, which might have been readily supplied, if not from the author's own experience, from the investigations of recent writers, more particularly those of MM. Louis and Bouillaud. We are glad to see that Mr. Mayo shews his acquaintance with the affection of the lining membrane of the heart which the last-named author has lately taken so much credit for noticing; but he might with advantage have treated the subject more at large than he has done, and have also vindicated the earlier claims of British medicine to the knowledge of the whole subject of rheumatic diseases of the organ. We must say a few words, in passing, on one observation which relates to the important subject of diagnosis. The author observes that "disease of the valves of the pulmonary artery is stethoscopically distinguished by its place and superficialness." The latter character may be admitted to be diagnostic, though its discovery must require no little nicety of ear. But it is an error to speak of the *situation* of the sound as distinctive of disease of these valves, since the same locality of sound must belong to the aortal valves, as the orifice of the one artery lies just behind the other. We have frequently noticed that young stethoscopists meet with a great many cases of that uncommon lesion, valvular disease of the right side, misled by their notions of what ought to be the respective seats of the sounds. The most frequent situation, and the greatest intensity of the bellows-sound is under the inferior third of the sternum, because the aortal valves lie just behind this spot; but it is so natural to fancy that disease in the left half of the heart must be heard considerably to the left of the sternum, that writers who might be supposed to know better have fallen into the error. Thus in that useful student's book, Martinet's Pathology, it is laid down that "if the left orifices are contracted, the bruit de râpe or bruit de soufflet is heard between the cartilages of the fifth and seventh ribs at the left side, whilst, if it occupies the orifices at the right side of the heart, the sound is most distinctly heard at the inferior part of the sternum." It is desirable that this twofold mistake of misplacing the sound of the aortal valves, and of attributing a bruit at the lower end of the sternum exclusively to disease of the right orifices, should be expunged from a work in such extensive use.

The section on the Veins is valuable and interesting. Subacute phlebitis is, in our author's experience, by no means a serious malady. The following extract illustrates the character of this inflammation and the practical use that may be made of it.

"I have in a considerable number of cases applied caustic or caustic paste over the trunks of the subcutaneous veins of the leg for varix. In some few instances, on the healing of the ulcer left by the separation of the eschar, no effect on the vein has been observable; but in much the greater proportion the vein has been found firm and hard, and its cavity obliterated at the part where the issue has been made. I have little doubt, that, in the successful cases, the irritation upon the vein has caused local subacute inflammation, as a consequence of which the blood has coagulated in its cavity, and plugged it. The vein is often tender, during several days, for the extent of three or four inches above the place at which the caustic is applied. The obstructed part does not exceed more than half an inch to an inch in length. I have never known acute phlebitis supervene in employing this practice." (P. 433.)

Respecting the origin of purulent depositions our author thus speaks:

"Of the attempts to explain the relation between acute phlebitis and the deposition and formation of matter in other parts, the most mechanical conjecture is, that the matter deposited in other parts is pus that has been first formed in the inflamed veins, and has thence been carried in the circulation to new places. The most original notion which has occurred to reasoners upon this question is, that the mixture of pus with the blood causes the blood as it circulates to form more pus; as the absorption of venereal virus causes a part to form more of the same poison. Gendrin's remarkable although inconclusive experiments, showing the rapid conversion of a clot into pus, favour this idea." (P. 435.)

The latter opinion was urged in a review of Dr. Carswell's "Elementary Forms of Disease," in the first number of this journal.* Mr. Mayo's own conjecture is as follows:

"The most cautious view may be to suppose, that, when one part of the serous surface of the vascular system is inflamed and suppurating, remote parts of the same surface—such as the capillaries of different organs—may with unusual readiness fall into inflammatory and suppurative action, and, without thickening the parts around, convert the blood circulating through them into pus" (P. 435.)

The section on the Arteries is likewise replete with information of the most valuable description. Whilst unprepared for therapeutical remarks in a treatise on Pathology so circumscribed as the present, we cannot consider intrusive in any work hints of really useful practice. Speaking of the means of arresting hemorrhage from a divided artery, the author observes,

"Pressure as a permanent means is rarely available, from the interruption of the circulation which it causes: but there are cases in which partial compression may be employed, and is found efficient. In hemorrhage from a deep wound of the palm of the hand, pressure may be directly applied to the bleeding vessel by means of a piece of sponge inserted into the wound, a raised compress being laid over it. If a similar compress is applied to the back of the hand, and a paper-knife laid upon

* Vol. I. p. 157.

each compress transversely to the hand; then by tying together the ends of the paper-knives on each side of the hand sufficient pressure may be made on the sponge in contact with the bleeding vessel to restrain the hemorrhage, without affecting the freedom of the general circulation in the part. Under this treatment, the wound inflames without much swelling, and suppurates; the extremity of the artery becomes plugged with effused lymph or coagulated blood, and the sponge becomes loose and comes away at the expiration of ten or twelve days. Bleeding after lithotomy is similarly stopped, by introducing a catheter through the wound into the bladder with lint fastened round it at the part, which is to lie against the plate of the ischium. Bleeding again from cysts of the thyroid gland injudiciously opened may be similarly arrested." (P. 440.)

The author has carefully described the changes in arteries effected by inflammation and perverted nutrition. The liability of the cerebral vessels to rupture from ossification is noticed, but no mention is made of one peculiar affection sometimes found in these vessels, which has not met with the attention it deserves. We allude to the formation of a solid deposit on their inner lining, which by the impediment to the circulation may occasion fatal apoplexy. This alteration we discovered in an elderly man who for many years had been subject to attacks of vertigo and coma, from which he recovered under the use of depletion, but who died at last with symptoms of apoplexy. There was no sanguineous or serous effusion, no ramollissement, or any alteration in the cerebral substance. The only morbid appearances were in the basilar and the anterior and middle cerebral arteries, which presented a great number of opaque patches, looking as if atheromatous matter had been deposited between the coats. But, on slitting them open, the opacity was found to be owing to nodules of a firm substance, resembling fibrinous concretions, either moveable or very loosely attached to the inner coat. Probably these bodies had at various times obstructed the circulation, not so much so however but that a diminution of the quantity of the blood by artificial means had been sufficient to abate the impediment for a time; the fatal attack must have been caused by a defective supply of arterial blood.

Of rupture of the arteries of the trunk the author has given two very interesting examples, one of which is remarkable for the simultaneous rupture of different parts of the aorta.

"A considerable ecchymosis (under the pericardium) was observed around the origins of the aorta and pulmonary artery, and about the right auricle. On slitting up the aorta from below, several patches of opaque, yellowish, and in some parts bony substance, appeared between the inner and middle coats. The aorta was large, and especially the first portion of the arch. The cellular membrane around the arch of the aorta contained clotted blood, by which also the external coat of the aorta was separated from the middle tunic, or at least from most of it, for a few circular fibres were attached to the interior of the cellular coat. This separation extended to the origin of the aorta, where it ended in a transverse slit in the inner and middle coats, immediately above the valve which has no corresponding coronary artery. No distinct opening could be traced into the pericardium from this slit, but in two or three places the membrane seemed so thin, that probably the aperture was concealed by the distention with blood of the cellular membrane beneath; or the blood may be supposed to have transuded. A similar, yet smaller transverse slit, appeared at the commencement of the arteria innominata. The sides of the left ventricle were enormously thick, and its cavity enlarged, it contained a small clot. The mitral valves very large and thickened at parts, but to no great extent. The auriculo-ventricular orifice large. All other parts of the heart healthy.

"An immense quantity of clotted blood was effused behind the peritoneum, along all the extent of the abdomen, aorta, and especially around the kidneys. On slitting up the aorta, two or three transverse slits were observed, similar to those mentioned to have been found in the thoracic vessels, with a like separation of the arterial coats." (P. 455.)

In the chapter on the Respiratory Organs, the reader will perceive that the author does not observe any strict similarity of plan in the discussion of his subjects. If he chanced to alight first upon the diseases of the membranes of the brain, he would have considered the work a very excellent compendium of pathological anatomy; but if, instead of this part, he had opened upon the diseases of the pleura, he would have taken it for a manual of practical medicine. It is impossible for us with our limits to follow the author step by step; besides that, an abridgment of an abridgment would be barren to the reader, and unjust to the writer.

Under the head of Diseases of the Pleura, we find nothing that requires particular notice, excepting a successful case of paracentisis thoracis, and an account of another, under treatment, of a less promising character. The former was a young subject; and of the unsuccessful instances of the operation which have fallen within our own experience none have attained an age above the middle. This fact has been noticed also by Baron Larrey, and may perhaps be accounted for not only by the greater buoyancy of constitution in such subjects, but also by the greater flexibility of the cartilages of the ribs, and their consequent readiness to adapt themselves to the shrunken contents of the chest. We do not find any notice of cartilaginous and osseous formations in the pleura, though they are by no means uncommon.

Our notice of the section on Diseases of the Lungs must be brief: like the others, it contains a good deal of valuable matter, but exhibits numerous oversights. Dilatation of the larger bronchi, an alteration of much importance with reference to the diagnosis of vomicæ, is not spoken of; contraction and obliteration of these passages are also unnoticed.

Mr. Mayo observes, that "ulcers of the larynx are of frequent occurrence, and are *often* combined with phthisis." We ask, are they ever combined with any other disease of a chronic nature, excepting syphilis? He adds, "Alone they constitute phthisis laryngæa." Do they ever occur alone, or, in other words, is this phthisis laryngæa ever independent of tubercles in the lungs on the one hand, or of a syphilitic taint on the other? We need scarcely remark that pathologists most capable of speaking to the question, MM. Louis and Andral, reply in the negative. It is possible but not proved that mercury may produce chronic ulcers of the larynx: the alleged instances are we apprehend of a mixed nature; that is, the metal had been administered in considerable quantity for the cure of syphilis.

The following is curious, though, as the author remarks, "not extremely infrequent."

"Mr. Charles Mayo, of Winchester, favoured me with a preparation from the body of an elderly lady, in which a firm whitish elastic polypus grew by a pedicle from the root of the epiglottis. It had produced occasional suffocative seizures, in one of which the patient died." (P. 514.)

The chapter devoted to the Digestive Organs begins with the morbid changes in the fauces, among which are the appearances of the tongue in different states of the system. Some of the more common of these are portrayed accurately enough, but, if the author thought it necessary to go into this department of semeiology at all, he would have done better to have treated it more amply. To exemplify some of the omissions: he mentions neither the patchy shining surface of the tongue, nor its turgid spongy condition, prominent papillæ, and salmon colour, so often observable in chronic mucous diseases complicated with tuberculous or carcinomatous cachexy; and (which is still more remarkable) no mention is made of the pale, sodden, indented tongue presented by chlorotic females, and sufferers from colonic dyspepsy. In a modern work, one is scarcely prepared to find hare-lip and congenital fissure of the palate dismissed without a hint of the improvement in our knowledge of these malformations since the researches of Serres, Isid. St. Hilaire, and others, upon the laws of organization. We should certainly be sorry to see any part of an elementary work taken up with the more fanciful and disputable theories belonging to this subject; but surely certain general principles respecting the arrest of development in different parts of the body and the analogy between morbid structures and inferior types of organization are as firmly established as any generalizations in the whole range of medicine.

Under diseases of the Œsophagus the author narrates some interesting cases of spasmodic difficulty of deglutition. In one it depended upon the irritation caused by ulceration of the interior of the larynx; in another it was probably sympathetic with derangement in other parts of the alimentary canal, and was cured by blue-pill and alteratives; in a third it appeared connected with gout; in a fourth, a lady under Sir B. Brodie's care, it was caused by anæmia, and cured by treatment directed against internal hæmorrhoids; and in the fifth it was complicated with "symptoms of inflammation of the peritoneal coat of the liver," and disappeared with the latter complaint. To these cases we are tempted to add one which occurred in our own experience not long ago. A young married woman had suffered dysphagia, which some practitioners considered structural, others spasmodic, for three years, during which time she had also been troubled with frequent and profuse hemorrhage from the uterus. On closely questioning her, we found that, although she had dated her illness from the first feeling of dysphagia, she had been subject to hemorrhage for many months before, and that this hemorrhage began during her recovery from a labour, of which an ignorant midwife had the charge, and in which great force was used in extracting the placenta. On examination per vaginam, we discovered an inverted uterus. It was almost impossible to help thinking of those deranged sensations in the gullet, particularly globus hystericus, so common in uterine disorders, and we were almost inclined to refer the case to a special sympathy between the vagina and œsophagus. But, on reflecting that between affections of the uterine system and such remote disorders as globus hystericus, there probably intervenes a peculiar state of the nervous system, that this state is often induced by other causes, and by none more frequently than by anæmia, and that spasmodic difficulty of deglutition has resulted, as in Sir B. Brodie's patient, from hemorrhage from other surfaces, we preferred concluding in the present instance that the inverted uterus was only indirectly connected with the stricture of the œsophagus. With the issue of the case we are unacquainted.

Stricture occasioned by corrosives is illustrated by a case of Dr. Wilson's, which the author witnessed in the Middlesex Hospital. A young woman swallowed about a tablespoonful of sulphuric acid on the 4th of January, and died of its effects on the œsophagus on the 14th of November.

The affections of the Stomach are discussed under the heads of hemorrhage, inflammation, indigestion from functional and structural causes, gelatinization, and malignant disease. With respect to the first of these we do not complain that the author says nothing of its diagnosis from hæmoptysis, or of its causes; but we cannot forbear remarking that he has expended much space on the semeiology of disorders certainly not more important than the present. Acute gastritis is treated of only as the effect of irritants, and the materials of this part are taken from Christison. Not a word is said of the difficulty and importance of distinguishing inflammatory injection from mere cadaveric congestion. It is true the profession, (thanks to the labours of Yelloly, Billard, Begin and Trousseau, Orfila and Andral,) are fully awake to the subject, but not more so than to the irritant action of arsenic, corrosive sublimate, &c.

Although acute gastritis may be rare as an idiopathic disease, it might have been as well to have devoted some little consideration to it as a complication of other diseases, thorough acquaintance with this subject being quite as valuable to the practitioner, as with the inflammation induced by poisons. Dyspepsia is examined in a very unsatisfactory manner. Some cases are related in which recovery took place; but whether they depended upon one and the same or upon different conditions of the stomach, whether chronic inflammation, or vitiated secretion, or morbid sensibility had most to do with them, are questions neither entertained nor hinted at; nothing occurs to remind one that such writers as Broussais, W. Philip, Barras and Johnson ever existed. Most of the cases are taken from Abercrombie. One got well by adopting a vegetable diet, another by means of sulphate of iron and aloes, a third by aloes only, a fourth by fresh-made curd, and a fifth by acetate of lead; but the student is left to guess why these agents were respectively apposite.

The following very judicious observations occur under the diseases of the duodenum.

"Vomiting is not so simple a consequence of duodenal as it is of gastric disease: it is here a symptom in some sort transitional between the effects of gastric and of intestinal disorder. Vomiting from duodenal disease either may be the result of irritation, like vomiting from affections of the stomach, or it may be the result of obstruction. The calibre of the duodenum is liable to be contracted by thickening of its coats to a degree which practically obliterates the cavity, when vomiting ensues, as in strangulated hernia. The duodenum closely adjoins the stomach, liver, and pancreas: it is situated before the spine, the psoas muscle, the right kidney, and ureter, and behind the ascending portion of the colon. There are few occasions for finer diagnosis than this complicated region affords. Disease can hardly exist in one of these parts without thorough contiguous sympathy implicating in a greater or less degree those adjacent to it. It is thus often extremely difficult in individual cases to identify the organ primarily attacked." (P. 311.)

Upon the much disputed theory of ileus the author thus confesses his faith:

"I am disposed to conjecture, that spasm and contraction of the muscular fibres of one portion of the intestine are the primary source of the disorder; the symptoms and the varied appearances of the dilated part of the intestine above being so much the same with those which are met with in different cases of fatal strangulated hernia. The remedies, which are beneficial in ileus, appear to me to countenance the theory of the disease which I have adopted. The principal of these are bloodletting, the tobacco injection, and opium, which might well allay and remedy spasm of one part, and a consequent state of distension, congestion, and inflammation of the portion above it; but are hardly calculated to relieve any supposed state of distension of a part of the intestine merely dependent upon weakness of its muscular structure. Dr. Abercrombie, from whose work on the Diseases of the Abdominal Viscera—rivalling, in scientific and practical interest, his Treatise on the Brain,—I have largely borrowed, is not disposed to admit the hypothesis of spasm; yet among his numerous illustrations of the subject there are many which seem to me to favour it." (P. 313.)

Our own belief is, that both spasm and paralysis are concerned in producing the obstruction. First, there is inflammation of the muscular coat, (whether beginning in this or propagated from the serous, we shall not enquire); 2dly, this inflammation causes spasm of the fibres, and if it did not, it would be an exception to muscular inflammation in every other part; 3dly, the morbidly contracted part refuses admission to the substances propelled from the superior portions of the gut; 4thly, the inflammation may be subdued by bleeding, and the spasm cease with it, or the latter may be more immediately overcome by a direct sedative influence, as that of opium and tobacco; 5thly, the inflammation may continue unchecked, in which case the fibres lose their contractility, (like the intercostal muscles in pleuritis, as explained by Dr. Stokes,) and though the commencement of the diseased part may receive solid matter from the healthy part immediately adjoining, it is incapable of propulsive contraction, so that the rest becomes distended by such substances as can find their way independently of muscular action, viz. air and liquids.

The constipation in peritonitis may, we think, be traced to a similar causation. An alvine evacuation in such cases is a favorable sign, not as proving the removal of an obstruction, but that inflammation has abated, and that the muscular fibres have consequently resumed their natural function, neither contracting too much nor too little; both extremes being, as we have shewn, incompatible with the propulsion of the fæcal matter.

The present section is concluded with a brief but vivid description of Cholera, some speculations upon its proximate cause which in the author's opinion is a derivation of blood to the stomach and bowels, and some pithy remarks upon the remedies employed. These are classed under the heads of empirical, rational, and paradoxical; calomel and bleeding belonging to the first, stimulants and opiates to the second, and cold affusion to the third.

We pass over the section on diseases of the Colon, as it contains little original matter. Of those of the rectum the first enumerated is the simple ulcer or fissure which occurs just above the sphincter. An interesting case is mentioned in which this affection simulated diseases of the prostate, but was cured by division of the sphincter and application of mercurial ointment to the ulcer. The *spreading* ulcer of the mucous with induration of the muscular coat, so prevalent among females in this country, is well described. The mucous membrane is often destroyed in this disease from half an inch above the anus to the sigmoid flexure of the colon, the muscular fibres become pale and atrophied, and the cellular membrane is hardened, though scarcely increased in thickness. The ordinary seat of *per-*

*manent* stricture the author has found to be from two inches and a half to four inches above the orifice of the gut; but his experience has not taught him to consider one part more subject than another to *spasmodic* contraction. He is of opinion that the latter kind of stricture is generally dependent upon vitiated secretions. We have met with it most frequently in hysterical females, in whom it has appeared and departed with the usual capriciousness of their other ailments. We cannot resist the temptation of extracting the remarks upon hæmorrhoids, as they certainly are multum in parvo.

"The different varieties which I have observed are,—1. Indurated inward piles, dark, firm, with little sensibility, protruding at each motion, with discharge of mucus. 2. Florid, soft, vascular protrusions of the mucous tissue in several folds, which bleed freely. These two kinds had better be tied, other circumstances being favorable. 3. One or more round blue knobs exactly at the margin of the intestine; therefore half covered by skin, half by mucous membrane. These should be let alone, unless one of them becomes extremely hard and distended and painful, when it had better be opened with a lancet. 4. Pendulous or ridgy folds of thickened skin around the anus, occasionally or constantly causing uneasy sensations, heat, itching, pain. These should be removed by the knife or scissors. 5. Warty excrescences occur round the anus, which should be removed with the scalpel, and the bases of each touched with caustic." (P. 353.)

There is nothing of particular interest in the section on Peritonitis. Ascites is not mentioned as an effect of chronic peritonitis. We are of opinion that the tubercular form of the malady has not received one half of the attention which it deserves, being far more frequent than would be supposed from the incidental manner in which it is spoken of in this and other systematic works. Its insidious origin and progress, its peculiar nature, and its constitutional relations demand for it the greatest nicety of management; and in but too many cases palliation is the utmost achievement of the practitioner.

Medullary sarcoma, melanoma, and gelatiniform sarcoma are mentioned as the malignant diseases of the peritoneum; and the forms and arrangement which the author assigns to each are nodules and clusters. He says nothing of that general thickening with hardening of the peritoneum, both visceral and parietal, which is not unfrequently found in combination with scirrhus of the liver; and which is most probably the effect of carcinomatous matter deposited in the subserous cellular membrane.

The section devoted to Hernia is very luminous in its descriptions and sound in its practical applications. With the cause of strangulation of the protruded part the author will not allow that spasm has any thing to do. We fully coincide in his belief that congestion of blood about the passage and neck of the sac may contribute to the constriction, but that the principal cause is "distention of the intestine with air and liquid; the traction so produced of the distended part upon the narrow aperture of the neck of the sac giving a tightness to the portion of intestine lying in it, which produces perfect obstruction." Many very valuable precepts upon the treatment of hernia are added.

The diseases of the liver are well arranged and for the most part accurately described; but we nowhere find any account of the fatty liver peculiar to tuberculous subjects. Certainly we cannot recognize it in the following paragraph, though the change here spoken of is the nearest approach to the lesion in question:

"*Steatosis of the Liver.* A substance resembling wax or adipocire is found in the liver, producing what on the analogy of a like disease in the voluntary muscles may be termed steatosis of that organ. This substance is sometimes found in irregular portions mixed with the healthy structure, and sometimes in small nodules like peas dispersed through the substance of the liver: in some cases the whole liver, or a large part, is found changed into an uniform mass of this appearance." (P. 400.)

This is very different from the fatty liver of phthisis, the characters of which our own observation has taught us to be, enlarged bulk, pale salmon colour, substance slightly brittle and granular, greasing the scalpel, rendering paper transparent, and yielding a considerable quantity of oil when toasted.

We must leave untouched the pathology of the spleen, pancreas, kidney, and genito-urinary system; and the whole chapter on the nervous system: this last is taken up in another article of the present number. No part of the work is better executed or appears to have more directly emanated from the author's own researches than the two first chapters which treat of the Bones and the Joints. A very clear account is given of the means whereby injuries of bone are repaired. The remarks upon reparation of the cranial bones and of the neck of the thigh bone within the capsule we think particularly satisfactory. The slowness of the process in the former instance is shewn to depend upon the want of a provisional callus, the fissure being filled up with osseous matter only by extension of bony growth from the opposite surfaces. The physical cause of the absence of a provisional callus is unknown, though the final cause, viz. the avoidance of pressure on the encephalon, is obvious. If the callus is furnished by surrounding tissues, certainly the scalp above and the dura mater below appear to be as little capable of contributing in this manner to bony reparation as any parts in the whole system. In the case of fracture of the neck of the femur, the surrounding tissues "are excluded by the untorn synovial and capsular membrane from communicating with the fracture."

Some interesting cases are related in the sections on hypertrophy and atrophy of bone. Under the head of inflammation of this tissue the author mentions two examples of inflammatory enlargement of the ribs,—one in a young lady whose health was not much impaired.

"The other had the following history. A young gentleman, through hard study and neglect of exercise, became dyspeptic, with pain in the head, and inability to collect his thoughts. After a time, three or four of his ribs on each side enlarged, and were painful. He gave up his studies, and went to the south of Europe, where he recovered. A year or two afterwards, he became a medical student in London. In a little time, his former indisposition returned; and he became again dyspeptic, with hypochondriasis, and painful swellings of the ribs. Upon going into the country, he recovered." (P. 25.)

We had a case recently in which three ribs on one side were inflamed and swollen. There were unequivocal marks of periostitis in other parts; the individual having been long subject to dyspepsia and rheumatism. Of abscess in bone the author has seen only one instance, but he alludes to three related by Sir B. Brodie in the 17th vol. of the Medico-Chirurgical Transactions.

The reader will find a careful description of the morbid processes in necrosis and caries, and the varieties of these affections well discriminated. The external characters of the malignant formations are given, but little is said of their minute structure and their origin. Upon this subject the author might have availed himself of Dr. Hodgkin's researches in the Medico-Chirurgical Transactions, without committing himself to the cystic theory.

We cannot afford space for noticing the Diseases of the Joints, the discussion of which, as of other departments of surgical pathology, the author conducts with even more than usual ability. We looked through the sections on the Voluntary Muscles and the Aponeuroses for some account of rheumatism. Respecting the former of these tissues, he observes that their inflammation "may be acute or chronic, circumscribed or diffused, or it may be rheumatic or gouty," (p. 124;) and the aponeuroses he tells us are "a principal seat of rheumatic inflammation." We are glad to detect in these passages a recognition of the principle that the essential nature of rheumatic and gouty inflammation is not determined by the seats which they occupy, though they evidently prefer some tissues to others. Mr. Mayo gives no opinion respecting the specific nature of these phenomena, and of their relation with certain constitutions, and more particularly with certain states of the blood.

The chapter on the Skin is divided into three sections, of which the first embraces those diseases which the tissue shares with other parts of the system; the second, the diseases peculiar to the skin; and the third "the principal forms of ulcers in which the skin is involved." The descriptions of the first two are borrowed principally from Rayer. "In the third, what I have stated is the result of my own observation, concurring, I suppose, with that of other surgeons." It is somewhat remarkable that on such a subject the author should not have had the curiosity to arrive at something more than a *supposition.* But a great deal of matter is condensed in this chapter which the student will find very useful as a collection of brief but characteristic descriptions.

Although the discharge of our critical duties has compelled us to point out many deficiencies in this work, it must have been noticed by those who have taken the trouble to follow us in our survey, that we have seldom had occasion to charge it with errors of statement or with falseness of reasoning; the freedom from which will compensate in a general view for many faults of omission. Considered indeed as a whole, these "Outlines of Pathology" form a very acceptable addition to medical literature; and no one, we venture to say, will regret having placed them in his library. Readers who open them with the expectation of finding traces of deep and extensive research, or of minute analysis,—expositions of comprehensive principles,—records of new observations, and original deductions, or such excellencies as distinguish the author's Outlines of Physiology, will scarcely fail to be disappointed; but we entertain not the slightest doubt that the hope expressed in the following extract from the Introduction will be more than realized.

"The author is not without hope, that the following work may be of service to students, if only by giving method to their enquiries, and by accustoming them to base the study of disease in anatomy, that it may be of help to those who visit or are engaged in forming pathological collections, that it may sometimes assist in the diagnosis of obscure cases by enabling the practitioner to review all the contingencies to which particular organs are liable; and may lead him, by the light again which method gives, to derive increased advantage from miscellaneous professional reading, as well as to tabulate and profit by, and to let others profit by, the results of his enlarging experience." (*Introduction*, p. xxviii.)

---

